# Frag-n-Fly gardening method for coral restoration - Pitching to the industry level the methods for coral fragmentation and outplanting in coral reef restoration

**DOI:** 10.1016/j.mex.2025.103602

**Published:** 2025-09-02

**Authors:** Bruno Welter Giraldes, Caroline Donahue, Eduardo Santos Mello, Hamad S. Al-Mohannadi, Syed Faisal Mustafa, Maryam Abdulla, Pedro Range

**Affiliations:** a Environmental Science Center, Qatar University, Doha, Qatar, POBox 2713; b Seascaping LLC, Subsea Eco-Engineering, Doha, Qatar; c Major Projects, Qatar Energy LNG, Doha, Qatar

**Keywords:** Climate change, Environmental compensation, Blue management, Blue engineering, Ecosystem restoration, In situ coral aquaculture

## Abstract

This study introduces the Frag-n-Fly method, an innovative approach to large-scale coral gardening designed to improve the efficiency of fragmentation and outplanting while meeting maritime industry standards. The method follows a six-step workflow implemented during two maritime expeditions and centers on a cache area adjacent to the restoration site, where scientifically validated artificial reef structures replace traditional land-based husbandry facilities. By eliminating terrestrial transport and aquaria-based acclimation, Frag-n-Fly reduces coral stress and provides a stable in situ environment for acclimation and fragmentation. Validation was achieved by processing 2000 colonies, producing over 20,000 fragments. The method demonstrated a fivefold increase in fragmentation throughput (∼50 vs. 10 colonies per hour), a ∼30–50% reduction in project costs, and early survivorship rates comparable or superior to traditional husbandry (0.11% vs. 0.69% mortality at 75 days). While Frag-n-Fly provides substantial gains in scalability and efficiency, it requires validated artificial reef technologies, specialized vessels, and trained personnel. The method is therefore best suited for industrial-scale, offshore restoration projects, while complementing traditional nursery-based approaches. Methodological advances:

• Coral gardening conducted entirely at sea, eliminating land-based logistics.
• Permanent cache areas with validated artificial reef modules ensure coral acclimation and survival.
• Industrial tools enable high-throughput fragmentation and large-scale cost-efficient outplanting.

## Graphical abstract

**Figure:**
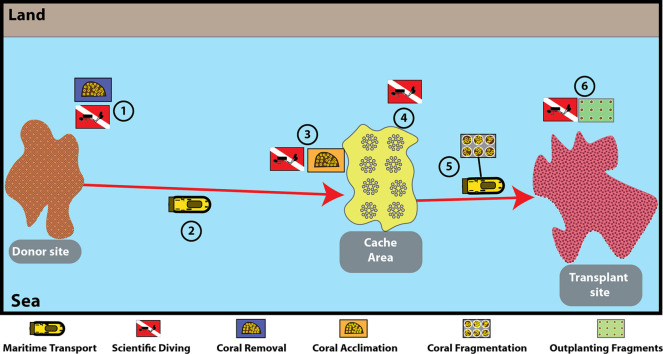
The proposed Frag-n-fly method using pernament table-like reef as a cache area.

## Specifications table

**Table:** 

**Subject area**	Environmental Science
**More specific subject area**	Coral Reef Restoration
**Name of your method**	Frag-n-Fly coral gardening method
**Name and reference of original method**	Name: Coral Fragmentation and outplanting methods; and Coral gardening methods. References Vaughan, D. E. (2021). Active Coral Restoration: Techniques for a Changing Planet. J. Ross Publishing, 636pp Boström-Einarsson, L., Babcock, R. C., Bayraktarov, E., Ceccarelli, D., Cook, N., Ferse, S. C., … & McLeod, I. M. (2020). Coral restoration-A systematic review of current methods, successes, failures and future directions. PloS one, 15(1), e0226631. Schmidt-Roach, S., Duarte, C. M., Hauser, C. A., & Aranda, M. (2020). Beyond reef restoration: next-generation techniques for coral gardening, landscaping, and outreach. Frontiers in Marine Science, 7, 672.
**Resource availability**	Cache area using artificial reefs with anti-sedimentation behavior; recommend the Mushroom Forest Artificial Reef modules [1,2]

## Background

Anthropogenic pressures are driving coral reef ecosystems toward collapse, severely depleting their economic and ecological functionality [[3], [4], [5]]. This is a special concern for coral reefs in the Arabian-Persian Gulf, a recognized marine area in biodiversity decline and considered as a natural laboratory for climate change effects as an extremely hot marine ecoregion setting [[6], [7], [8], [9]]. The rapid expansion of these collapsing resources, combined with the dwindling number of pristine coral reefs, has spurred significant efforts to restore these highly productive ecosystems [[10], [11], [12], [13], [14], [15], [16], [17], [18]]. Recent advancements in coral restoration methods and technologies are promising; however, most restoration approaches are still research-oriented and not optimized for large-scale applications [19].

From an industrial perspective, coral restoration efforts remain in the early stages of technological development, often rated as low on the Technology Readiness Level (TRL) scale. This limits their suitability for large-scale environmental offset programs, such as those required by industries with substantial environmental footprints, including the Oil and Gas sector [[20], [21], [22]]. To meet the growing demand for large-scale restoration, industrial methods for coral gardening must be developed, delivering results efficiently and reliably while adhering to established regulatory standards.

This study introduces a validated method designed for large-scale coral gardening, tailored to meet the rigorous requirements of the Oil and Gas industry. This is an important management tool, particularly in a scenario where natural rewilding is often insufficient to restore the productivity of degraded blue resources [[23], [24], [25]] and where active restoration strategies have become indispensable. For this active restoration, adding economic value to restoration actions through stakeholder collaboration as a Blue Management practice [24] has emerged as a practical solution. This approach was implemented in this study as part of a large project funded through an environmental offset program by the Oil and Gas industry, where researchers at Qatar University’s Environmental Science Center (ESC) developed a series of experiments aimed at advancing coral reef restoration technologies in the Arabian Gulf. This effort was part of the Coral Management Plan for Qatar Energy LNG’s North Field Expansion (NFE) and North Field Production Sustainability (NFPS) projects.

In this project, 2000 coral colonies required relocation, providing an opportunity for research and methodological improvement. The first 1000 colonies were processed using a traditional husbandry-based method using a land-based aquaculture facility for acclimation and fragmentation. Lessons learned from this process highlighted inefficiencies in logistics and scalability. To address these challenges, the Frag-n-Fly method was developed to optimize coral gardening on an industrial scale [18]. The remaining 1000 corals were allocated for testing the Frag-n-Fly methodology. A cache area was established using the Mushroom Forest Artificial Reef (MFAR) [1], a technology proven for stability and sedimentation resistance [2]. This innovative approach aims to replicate the efficiency standards of terrestrial agricultural systems while minimizing time, cost, effort, and environmental impact. In summary, this study presents the Frag-n-Fly method as a novel, scalable, and efficient solution for large-scale coral restoration, offering a significant advancement in the field of coral gardening and ecosystem restoration*.*

## Method details

### The Frag-n-Fly coral gardening overview

**Fig. 1:**
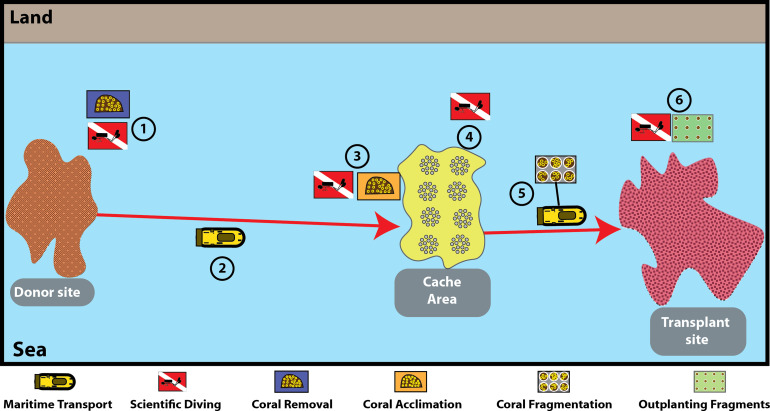
The Frag-n-Fly coral gardening method, including the reference areas (Donor site, Cache Area, and Transplant site) and the 6 steps from colony removal to outplanting the fragments.

The Frag-n-Fly method (Fig. 1) involves six steps divided into two maritime expeditions. During the first expedition, three steps are carried out: Step 1 - Coral removal from the donor site, performed by skilled and trained divers; Step 2 - Transportation of the corals in specially adapted boats to minimize stress caused by the initial removal; Step 3 - Placement of the corals in the cache area for an acclimation period, also carried out by trained divers.

In the second maritime expedition, the remaining three steps are performed: Step 4 - After the acclimation period, the corals are removed from the cache area and loaded onto the vessel; Step 5 - Onboard the vessel, the fragmentation process begins immediately after coral removal, along with the preparation of cement; Step 6 - The cement and coral fragments are deployed in the water near the “coral garden area” at the transplant site. The fragments are then permanently affixed to the natural hard substrate at the site using cement.

### Preliminary actions

**Fig. 2:**
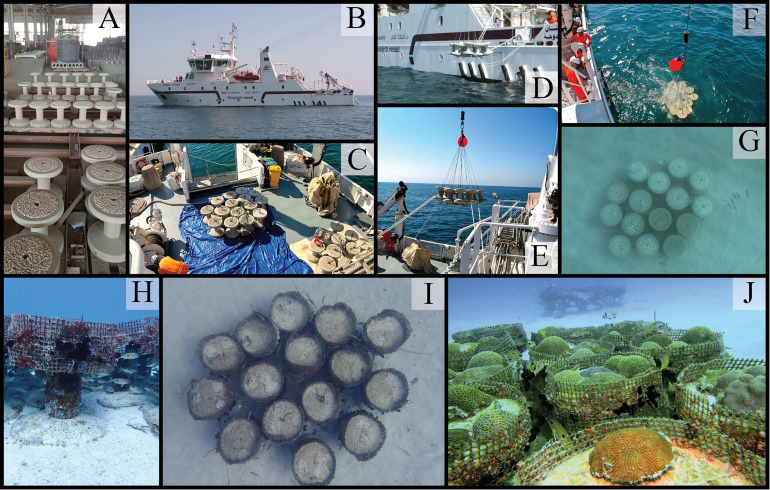
The subsea installation of the Mushroom Reef to build up the Cache Area, with (A) the units cast on the precast factory, (B) the R/V Janan, (C) the units assembled in packs on the R/V deck, (D, E, F) the deployment of the packs, (G) the pack installed underwater, (H) the mushroom units with the nets on top, (I) the cache with nets ready to receive the corals, (J) the coral placed on the cache area.
• Preparation of the transplant site - At the transplant site, the gardening area must be marked underwater using pins and defined with GPS coordinates. It is crucial to select a section of hard substrate without dense live coral coverage. In addition, an underwater map of the gardening area should be prepared, and an outplanting strategy must be defined. The recommended placement strategy is linear (along a transect) to facilitate the underwater monitoring; however, square or circular layouts can also be used depending on the seascaping (underwater landscaping) strategy.

**Fig. 3:**
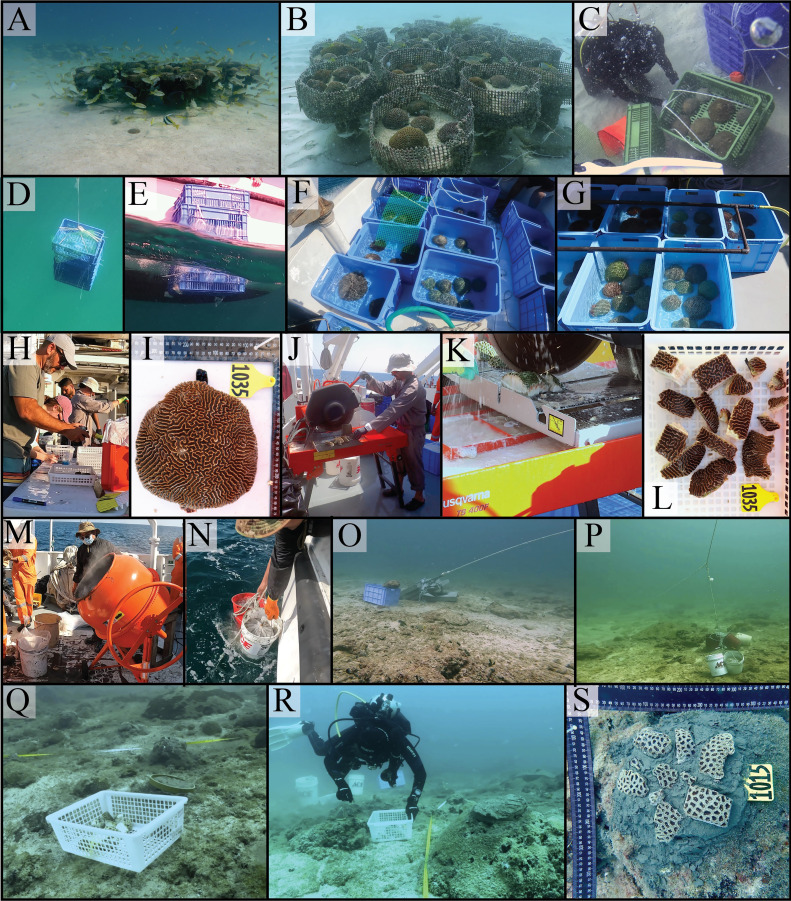
The process of collecting corals from the temporary cache area, then fragmenting, tagging, and outplanting via the Frag-n-Fly Method.
• Concrete Mixer - This machine onboard the vessel is essential in the proposed method for preparing a large amount of cement in a short period, allowing prompt deployment and readiness in the coral gardening processes (Fig. 3M).
• Coral Acclimatization Tanks - These are water tanks with constant seawater circulation (Fig. 3F and G) and easy access (e.g., short lateral sides). They are vital for managing coral colonies and fragments during transportation onboard the vessel. Multiple small tanks are recommended to create isolated “aquarium microcosms,” each holding a few corals, to avoid cross-contamination in case of diseases or excessive coral mucus (a stress response and/or defense mechanism).
• Transportation boxes - These plastic storage crates can be stacked and securely closed, and they are used for lifting and deploying coral colonies and fragments from the boat to the sea bottom and vice versa (Fig. 3C–E).

• Evaluation of the donor site - An environmental assessment of the donor site is essential to identify the main coral composition, the distribution of colonies on the seabed, and the health status of the corals intended for use in the gardening process. Based on the number of colonies planned for removal in the project, it is possible to calculate the required hours and the number of divers necessary to remove all colonies.
• Subsea installation of the cache assets (Fig. 2) - The installation of cache assets involves several steps: 1 - Select a soft-bottom area near the transplant site to serve as the cache area, ensuring it is close enought to facilitate the coral outplanting logistics, but far enough to avoid affecting the natural reefs. (the site should ideally be chosen using hydrodynamic modeling based on local current patterns to minimize the risk of sedimentation from the artificial reefs); 2 - Prepare a subsea engineering project based on the hydrodynamic patterns of the selected site, including the designs for the artificial reefs; 3 - Cast the artificial reefs following the subsea engineering project and designs; 4 - Deploy the artificial reefs at the selected site using a vessel equipped with a crane (Fig. 2D–F). It is important to choose table-like artificial reefs to facilitate the placement of corals and structures with efficient hydrodynamic performance to prevent sedimentation on the coral colonies during the acclimation period. We recommend the use of the Mushroom Forest Artificial Reef (Fig. 2A) due to its validated hydrodynamic performance [2]. This was the model used in our cache area when the proposed Frag-n-Fly method was developed. Additionally, we suggest attaching a net to the lateral tops of the Mushroom reefs (Figs. 2H–J and 3A and B) to hold the corals during the acclimation period without requiring cementation.

### Required materials and personnel

A list of equipment and personnel is essential for successfully implementing the proposed Frag-n-Fly method.

#### Required equipment

• Adapted Boat/Vessel - The vessel is used at various stages and must include: A crane for deploying the artificial reefs; A water circulation system to replace water in the aquariums/tanks where the corals are placed; A deck area for conducting the fragmentation and cement preparation; A diving structure to facilitate the deployment of cement and coral fragment boxes at the diving site and to support diving activities. In the present experiment, the Research Vessel Janan from Qatar University was utilized (Fig. 2B), however, alternative vessels such as barges, speed boats and/or dive boats can be used.
• Industrial Stone (Masonry) Saw / Tile Cutter - An industrial tile cutter with a seawater circulation system demonstrated to be functional and effective for cutting hard scleractinian corals quickly. The seawater circulation hydrates the soft tissue of the coral during the cutting process (Fig. 3J and K).

#### Required personnel

• Aquarists/Biologists Experts - A biologist/aquarist with expertise in coral husbandry is essential for managing onboard activities. Their responsibilities include: validation of species identification; risk assessment (e.g., treating diseased corals); overseeing the fragmentation process to minimize damage to the soft tissue of scleractinian corals; and monitoring fragment size and health for each coral species.
• Skilled Divers - A trained team of divers is required to select coral colonies and species, and to handle corals carefully during removal, lifting, deployment, and outplanting (gardening). Depending on the maritime regulations of the region or industry (e.g., Oil and Gas standards), either scuba divers or technical divers may be employed.
• Subsea Engineer - A skilled subsea engineer is needed to prepare and manage the subsea engineering installation project for the cache area. Their responsibilities include the preparation of the blueprint design of the artificial reefs (considering hydraulic/hydrodynamic concerns), casting the artificial reefs, and ensuring the proper subsea installation of the cache assets on the pre-selected seabed.

### Detailed action per step

#### Expedition 1

**Step 1.** Entire coral colonies are removed from the seafloor using crowbars, hammers, and stone chisels. The corals are detached from the hard substrate at their base, avoiding contact with or damage to the external/superior soft tissue of the colonies. The corals are then carefully placed in transportation boxes and lifted to the sea surface using diving lifting bags.
**Step 2**. After retrieving the corals from the sea surface, each coral is placed individually into acclimation tanks onboard the vessel. These tanks must have a constant seawater inflow to reduce stress and flush out water containing coral mucus. With continuous water circulation, the corals are transported onboard the vessel to the cache area.
**Step 3.** Upon arrival at the cache area, the corals are removed individually from the acclimatization tanks and placed into transportation boxes. The boxes are then deployed to the seabed near the artificial reefs. On the seabed, divers carefully remove the corals from the transportation boxes and place them atop table-like artificial reefs without cementation (Fig. 3A and B). The corals are then able to safely acclimatize to the water conditions virtually identical to the transplantation sites and can remain at the cache area until the second expedition.

#### Expedition 2

**Step 4.** The coral colonies are retrieved from the cache area; they are then placed in the transportation boxes and lifted to the sea surface using diving lifting bags (Fig. 3C–E). Once onboard the vessel, the corals are placed into acclimation tanks (Fig. 2F and G). As before, the tanks must have a constant seawater inflow to minimize coral stress and flush out water containing coral mucus.
**Step 5.** During maritime transportation from the cache area to the transplant site, the coral colonies are fragmented. Each colony is removed from the acclimation tanks and fragmented using the industrial masonry saw (Fig. 3H–K). The optimal fragment size varies depending on the coral species. Once fragmented, the coral pieces are placed into trays, grouped by their parent colony (Fig. 3L), and returned to the acclimation tanks. After fragmentation is complete, cement preparation begins. Using a concrete mixer and pure Portland cement, the cement is prepared (Fig. 3M) using an adapted mortar concept previously described in references [26].
**Step 6.** Just before the coral outplanting/gardening process, trays with coral fragments are placed into transportation boxes. Plastic bags filled with prepared cement are then placed into buckets. These transportation boxes and buckets are deployed to the pre-selected gardening area (Fig. 3O and P). Once at the gardening area, the buckets and transportation boxes are distributed according to the predefined outplanting strategy. Templates are strategically placed on the seabed to mark the exact locations for planting the fragments (Fig. 3Q). Hard substrate areas with minimal benthic coverage or barren patches are selected. The template shape determines the final shape of the coral colonies, as the fragments grow and fuse over time to cover the cemented area. For this project, PVC pipe rings (5 cm in height, 20 cm in diameter) were used as molds, resulting in corals with a circular shape. After placing the molds, cement dough is filled into each mold, and coral fragments are carefully embedded in the dough (Fig. 3Q, R and S). It is critical to handle the fragments gently, ensuring the living soft tissue remains upright and above the cement.

### Industrialization level in coral gardening

To improve industrialization in coral gardening, it is essential to optimize the production line, reduce efforts and costs, and increase efficiency. Several improvements were achieved in the proposed Frag-n-Fly method, including:

• **Excluding the Land-based Husbandry** - In many coral gardening projects, including this one initially, coral acclimatization and fragmentation are conducted in husbandry facilities. By excluding the husbandry step, industrial improvements were achieved, including: 1) Reduction in coral transplantation logistics, completely eliminating the need for terrestrial transportation; 2) Decreased effort in maintaining aquarium facilities during coral acclimatization. This significantly mitigates issues such as coral mucus production, disease control, and algae proliferation. In the cache area, the corals are exposed to water conditions identical to their natural habitat, with the presence of functional species that clean/protect corals on nature. In summary, excluding the husbandry stage of coral gardening increases the logistic efficiency, reduces the coral stress and chances of mortality, and reduces the cost of maintaining costly aquaculture facilities and aquarist experts in corals (expensive workforce and facility).
• **Reducing the number of Expeditions** - The method requires only two maritime expeditions, aligning better with the restricted schedules of maritime activities involving vessels and divers. This is a significant advancement for environmental offset programs within the Oil and Gas industry. The first expedition allows the removal of a large number of corals in a single operation (with divers and underwater time calculated per number of corals aimed to be removed). The corals, acclimated in a cache area near the transplantation site (the natural reef to be restored), can be transplanted during a second expedition, which can be scheduled as needed, and the length and number of subsequent expeditions can be flexible depending on the number of fragments planned for gardening. Therefore, the reduced number of required expeditions increases the efficiency and reduces the costs of the coral gardening activity. Highlighting that one of the main limitations for performing maritime activity is the limited number of good weather periods per year, and therefore with only 2 expeditions required for coral gardening represents a massive logistics improvement on the entire process.
• D**iving activity** - Using a cache area to host the corals enables the removal of many colonies in a single day of diving activity (Expedition 1). In the land-based methods, the limited space in husbandry facilities constrained the number of corals that could be collected per diving session (proportional to the available space in the aquariums). In the Frag-n-fly method, the number of corals removed is limited by the avalable space in the cache area., Highlighting that the installation of the cache area (artificial reefs) didn't require divers. However, the "Frag-n-Fly" method requires more diving activities for placing and removing the corals from the cache reefs.
• **The use of a cache area** - The cache area significantly reduces costs related to expensive aquarium maintenance in husbandry facilities, which demand intensive upkeep and specialized labor (aquarium experts). Additionally, ecological and environmental acclimatization is improved in the cache area, as the water conditions closely match those of the transplantation site. The cache area also allows natural marine life assimilation, including functional species, such as herbivores and coral-cleaning organisms, which maintain coral health naturally as in ecosystems. The cache area size can be adjusted to accommodate the intended number of corals. In this project, the installed artificial reef accommodates 500 coral colonies (10–20 cm in diameter) and costs 30 % of the total cost of installing and maintaining the aquarium facility.
• **The fragmentation process** - Using an industrial masonry saw for coral fragmentation significantly increases productivity and reduces processing time, allowing fragmentation of approximately 50 colonies per hour. In this study, photographing all colonies and fragments for research purposes slightly extended the process, but without this step, additional colonies could be fragmented per hour. Another improvement is the "Frag-n-Fly" method onboard the vessel, where fragments are immediately transported to the outplanting site after fragmentation, accelerating the overall process.
• **The outplanting/gardening process** - The industrialization status of the proposed outplanting/gardening process focuses on efficiency and the large number of fragments that can be transplanted per dive. With ample cement distributed across the transplant area, four divers can outplant 100 colonies or 1000 fragments per hour (assuming that each 10–15 cm diameter coral colony produces approximately 10 fragments).

## Method validation

**Fig. 4:**
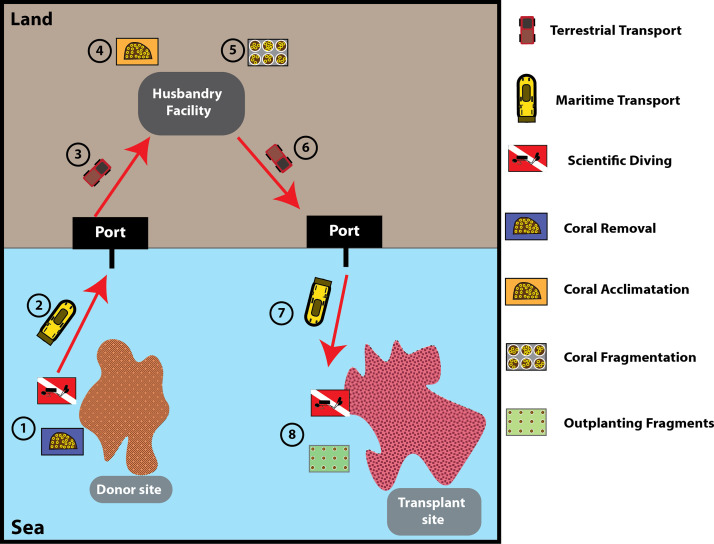
Land-based husbandry fragmentation process (“original method”) used prior to the development of the Frag-n-Fly method.

In the project where the Frag-n-Fly method was developed, a total of 2000 coral colonies had to be removed as part of an environmental offset program. For the first 1000 colonies, we used the “original method,” which involved establishing a land-based aquaculture/husbandry facility. Colonies were transported by sea and land to the facility, fragmented under controlled conditions, and subsequently transported again by land and sea for transplantation at the restoration site (Fig. 4). Due to the limited aquarium space, the entire process had to be divided into five batches of 200 colonies, meaning that the full cycle illustrated in Fig. 4 was repeated five times. Although this method enabled acclimation and fragmentation under controlled conditions, several logistical and efficiency limitations became evident.

**Fig. 5:**
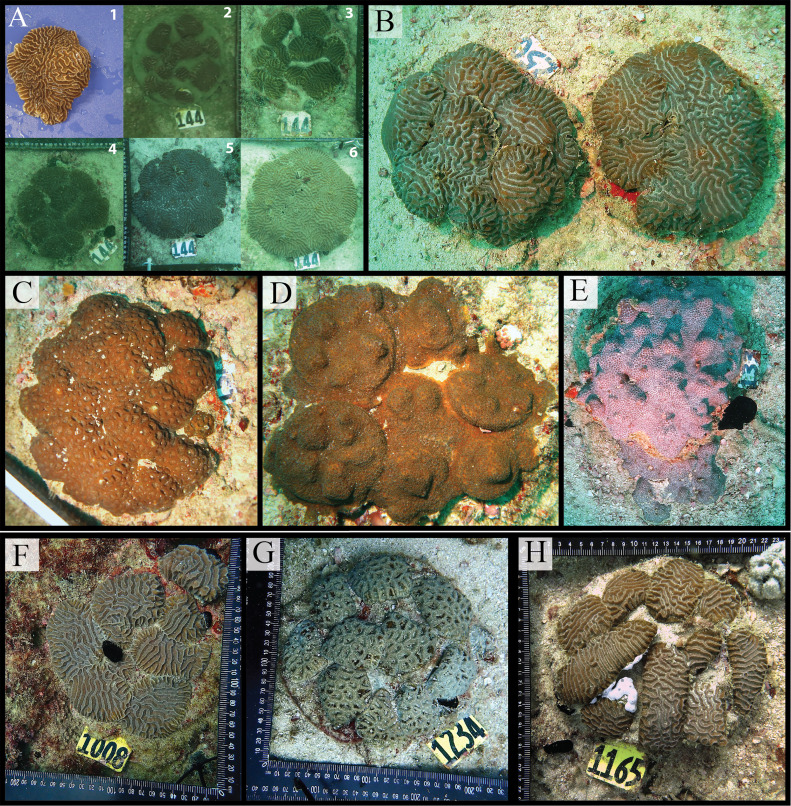
The validation of coral gardening methods and the growth of coral colonies after outplanting. Using the Land-based method: (A) a time series of a *Platygyra daedalea* (Ellis & Solander, 1786) (colony 144) after outplanting, with the original colony [1], after outplanted [2], and progression of growth from the first monitoring (219, 336, 723, and 1067 days after outplanted [3,4,5,6 respectively]). Other coral species with similar complete fusion growth after outplanted; (B) *P. daedalea* in two outplanted units (1067 days); (C) *Dipsastraea pallida* (Dana, 1846) (1067 days); (D) *Cyphastrea microphthalma* (Lamarck, 1816) (600 days); (E) *Cyphastrea* cf*. serailia* (Forskål, 1775) (1067 days). Using the Frag-n-Fly method (F) *P. daedalea* after 258 days; (G) *D. pallida* after 185 days; and (H) *P. daedalea* after 140 days.

Based on these lessons, we developed the Frag-n-Fly method as a more industrialized approach. Validation was performed with the remaining 1000 colonies, divided into two batches of 500 each, and held in a cache area designed to accommodate 500 colonies. The primary validation criterion was coral survival. During acclimatization, survival was comparable between methods, with <3 % mortality in both husbandry tanks and the cache. Post-fragmentation and transplantation survival is being evaluated in a separate long-term study. Preliminary monitoring at 75 days—the most critical period for coral gardening—showed 0.69 % mortality under the husbandry method compared to only 0.11 % with Frag-n-Fly. Growth rates were also markedly improved, with several colonies showing >100 % increase in size within one year (Fig. 5). Overall, more than 20,000 fragments were produced and outplanted. While both methods yielded high survival, Frag-n-Fly demonstrated clear gains in efficiency and scalability.

### Coral stress

A key limitation of the land-based method (Fig. 4) is the series of stressors imposed due to the logistics. Immediately after detachment, corals undergo a physiological stress response characterized by the excessive production of mucus [27,28], a defense mechanism that requires constant water renewal to avoid smothering of colonies and deterioration of water quality. This becomes particularly problematic when vessels enter polluted coastal waters or ports, where water exchange is limited and additional sea water reservoirs are required to maintain quality. Stress increases further during terrestrial transport, where water renewal is limited and accumulated mucus can rapidly cause mortality during long transfers.

Upon arrival in aquaculture facilities, corals are abruptly exposed to water conditions that differ from their natural habitat, prompting further mucus release. While small-scale operations can mitigate this through skimming and filtration, large number of corals (as used here) quickly overload filtration systems, leading to deteriorated water quality and elevated stress. Corals also require artificial feeding regimes, which may be nutritionally unbalanced, increasing disease susceptibility. Finally, after fragmentation in aquaria, colonies again produce mucus in large volumes, intensifying the burden on filtration systems and workforce requirements.

By contrast, the Frag-n-Fly method (Fig. 1) minimizes these stressors. Corals acclimate in cache areas with environmental conditions similar to the transplant site, where natural hydrodynamics provide continuous water renewal, dispersing mucus without filtration overload. Fragmentation and post-fragment recovery occur in situ, allowing corals to heal under natural conditions that better support resilience. This reduces cumulative stress, mortality risk, and husbandry effort, making the method more scalable for large-scale restoration.

### Duration of each method

The total time required differed substantially between methods (Table 1). The Frag-n-Fly method required six steps: (St1) coral removal [1 day], (St2) maritime transport [1 day], (St3) cache deployment [1 day], (St4) colony retrieval [1 day], (St5) vessel-based fragmentation [1 day], and (St6) outplanting [2 days] (Fig. 1). For 1000 colonies, this equated to 7 expeditions (2 for St1–3 + 5 for St4–6), totaling 26 working days (3 days x 2 expeditions + 4days x 5 expeditions).

**Table 1:** Comparison of operational time between methods for processing 1000 coral colonies.

	Land-based method	Frag-n-Fly method
**Acclimation facility**	1–3 years for the aquaculture facility installation	30 days for casting the reefs and 2 days for the subsea installation
**Action steps**	8	6
**Required number of Marine Expedition** (allocation of personals and facility)	10	7
**Total Action days**	55	26
**Fragmentation rate**	10 colonies/h	50 colonies/h

The land-based method involved eight steps: (St1) coral removal [1 day], (St2) maritime transport [1 day], (St3) terrestrial transport [1 day], (St4) organizing husbandry acclimation [1 day], (St5) fragmentation [3 days], (St6) terrestrial transport [1 day], (St7) maritime transport [1 day], and (St8) outplanting [2 days] (Fig. 4). For 1000 colonies, this required 10 expeditions (5 for St1–4 + 5 for St4–8), totaling 55 working days (5 days x 4 expeditions + 6 days x 5 expeditions).

The coral acclimation time required for both methods (in the cache area or in the land-based husbandry) depends on the availability of resources, facility, weather, among other variables, and can take from few days to several months. Therefore, this resting/healing period can be adjusted as required. The main difference is that the acclimation time on the cache area has no personal and maintenance costs, such as those required for the land-based husbandry facility.

### Cost and efforts

Cost comparison (Table 2) highlights the efficiency gains of Frag-n-Fly. Eliminating the land-based husbandry stage reduced expenses associated with construction, daily labor, feeding, and filtration, which accounted for 60–85 % of total costs in the land-based method. Although cache deployment introduced new expenses (5–15 % of total costs), these were offset by the elimination of husbandry facilities. Vessel requirements per expedition were higher for Frag-n-Fly, but the reduced number of expeditions lowered total vessel days by ∼30 %, cutting costs and risks. The use of industrial masonry saws increased fragmentation throughput fivefold, further improving cost-effectiveness. Overall, Frag-n-Fly can reduce total project costs by ∼30–50 %, consistent with previous cost-efficiency analyses of coral restoration [29].

**Table 2:** Comparative cost and effort analysis of the land-based husbandry method versus the Frag-n-Fly method, expressed as relative costs and effort for processing 1000 coral colonies.

Category	Land-based method	Frag-n-Fly method	
**Aquarium / Husbandry Facility**	Requires construction/maintenance of a high standard aquaculture facility, seawater systems, pumps, food, disease control, daily labor; ∼60–85 % of total costs.	Not required; cache area substitutes with passive in situ acclimation.	↓ Eliminates major capital & maintenance costs.
**Cache Area / Artificial Reefs**	Not required.	Requires subsea engineering, precast artificial reefs, and deployment (2 expeditions); ∼5–15 % of total costs.	↑ New cost, but offset by facility savings.
**Vessel Operations** (expedition costs)	10 expeditions	7 expeditions	↓ 30 % less vessel usage.
**Diver Effort** (same number of divers)	5 dive operations (coral removal). 5 dive operations (coral outplanting). Totaling 10 dive operations.	2 dives (coral removal), 2 dives (placement on cache), 5 dives (removal from cache), 5 dives (coral outplanting. Totaling 14 dive operations.	↑ 30 % more dive activity.
**Specialized Staff**	Continuous aquarist biologist labor for weeks/months.	Requires aquarium expertise only during expeditions.	↓ Lower personnel costs.
**Fragmentation Equipment**	Bandsaw in laboratory; slower throughput (∼10 corals/hour).	Industrial masonry saw on vessel; faster throughput (∼50 corals/hour).	↑ Productivity × 5.
**Logistical Complexity**	Synchronization of land + sea transport; coral transfers between aquaria, boats, reef.	Vessel-based operations only; no land transport required.	↓ Simplified, fewer steps.
**Overall Cost (per 1000 colonies)**	100 % (baseline).	∼50–70 % of baseline (≈30–50 % cost reduction).	↑More cost-efficient at scale.

### Success metrics

To systematically compare both approaches, a success matrix was developed (Table 3). Evaluation across time, cost, effort, productivity, quality, and risk confirmed that Frag-n-Fly achieves higher throughput and scalability, while the land-based method remains more logistically demanding and cost-intensive due to aquaculture facilities.

**Table 3:** Success matrix comparing the Frag-n-Fly method with the land-based husbandry method across performance dimensions.

Success Matrix Topic	Frag-n-Fly Method	Land-based Method
**Time**	7 expeditions; 26 days for processing 1000 corals (Excluding acclimation days)	10 expeditions; 51 days for processing 1000 corals (Excluding acclimation days)
**Cost**	∼30–50 % lower total cost; no aquaria facility required; savings in labor, vessel time, and infrastructure; cache setup adds some upfront cost.	Higher total cost due to coral aquaculture facility construction and maintenance, continuous labor, daily feeding, disease control, electricity, filtration, and multiple vessel operations.
**Effort**	Streamlined logistics (; fewer qualified staff required overall; natural seawater circulation reduces maintenance burden. Higher diving required	Labor-intensive; requires aquarium biologists, technicians, daily maintenance, and multiple teams for land-sea coordination.
**Productivity**	High throughput: ∼50 colonies/hour fragmented with industrial masonry saw; Faster coral gardening process in general.	Lower throughput: ∼10 colonies/hour with bandsaws in lab; slower outplanting due to synchronized land-sea logistics.
**Quality**	Natural seawater conditions during acclimation and healing reduce coral stress and mucus accumulation. Can be risky on selecting the wrong cache area.	Controlled condition. Despite the chances of overloading the filtration systems with large scale-productivity, on aquariums the corals can be protected from intense summer heats. Diseases can be treated.
**Risk**	Lower cumulative stress by avoiding terrestrial transport and artificial acclimation; reduced maritime expeditions lower exposure risk; cache areas still vulnerable to fishing gear or environmental stressors.	Elevated stress from transport, artificial diets, and repeated handling; higher risk of disease in crowded aquaria; maritime expeditions days increase operational risk.

## Limitations

The Frag-n-Fly method represents a significant advancement for large-scale offshore coral gardening (above 1000 colonies), but its implementation also introduces unique constraints that differ from traditional land-based or small-scale in situ approaches. These limitations are primarily linked to environmental dependence, cache area design, artificial reef technology, logistical demands, species-specific responses, and regional capacity. Recognizing and mitigating these constraints is essential to optimizing the method for broader applications in global coral restoration.

### Environmental and climatic constraints

As with all coral gardening methods, Frag-n-Fly operations remain dependent on stable environmental conditions. Critical steps such as coral removal, acclimation, and outplanting require favorable weather windows. Although the method reduces the total number of expeditions compared to land-based husbandry, prolonged storms or rough seas may delay operations and increase risks. Similarly, cache and transplant sites must be carefully selected to avoid areas with turbidity, pollution, or thermal extremes, as such conditions can compromise coral survival. Unlike aquaria, cache areas cannot buffer environmental variability and therefore require strategic site selection and scheduling of opperations.

### Cache area vulnerability and artificial reef technology

The reliance on temporary cache areas presents distinct challenges. Corals placed in cache modules are exposed to external threats such as fishing gear, boat anchoring, or sedimentation. Biological risks, including bleaching or disease, may also be higher in regions with poor water quality or pathogen prevalence.

A critical factor in mitigating these risks is the selection of appropriate artificial reef technology. Unvalidated structures lacking hydrodynamic efficiency can trap sediments and cause coral mortality during the acclimatization period. Moreover, poorly engineered modules may alter local flow regimes, creating sediment plumes that extend for several kilometers and compromise natural reefs at transplant sites [29,30]. For this reason, we emphasize the need for reef technologies with validated hydrodynamic performance, sediment-avoidance properties, and proven coral survival outcomes. In this study, we selected the Mushroom Forest Artificial Reef (MFAR), a design validated for hydrodynamic efficiency and sedimentation control [2], and subsequently confirmed as effective for coral survival and farming in cache applications. The use of MFAR modules ensures that cache areas function as intended, providing safe acclimatization environments that minimize sediment stress while maximizing coral survivorship.

### Logistical demands

Although the Frag-n-Fly method streamlines the overall restoration workflow, it requires access to specialized maritime and technical infrastructure. Research vessels or workboats with deck space, lifting gear, and industrial masonry saws are essential to carry out large-scale operations. Skilled scientific/technical divers are required for coral removal, placement, and outplanting onboard vessels. In addition, establishing cache areas requires subsea engineering expertise, precast reef fabrication facilities, and port access. These requirements may limit applicability in regions lacking advanced maritime infrastructure or trained personnel.

### Species-Specific considerations

It is unknown if there is a species-specific relation on using the Frag-n-Fly method with a cache area. The method may not be equally effective for all coral taxa. It is expected that robust and resilient species, particularly those adapted to disturbance and climate variability used in this study, might perform better using this approach. However, fragile, rare, or slow-growing species may require a different approach. In such cases, alternative strategies such as land-based nurseries, assisted evolution, or selective propagation may be considered. Thus, species choice is a critical factor when applying Frag-n-Fly at scale and the list of species validated for using this method need to be presented in further studies.

### Applicability to remote or low-infrastructure regions

Implementation of Frag-n-Fly requires initial investment in reef modules, vessel operations, specialized equipment, and trained staff. These requirements may pose barriers in remote or underdeveloped regions where maritime logistics and technical expertise are limited. By contrast, small-scale land-based or in situ nurseries may be more practical for community-led projects. Frag-n-Fly is therefore best suited for industrial-scale, offshore, or infrastructure-supported projects, where its efficiency, scalability, and stress reduction benefits outweigh the higher initial investment.

## Conclusion

In summary, the Frag-n-Fly method is not a universal substitute for all coral restoration approaches. Its greatest strengths lie in industrial scalability and operational efficiency, but it is constrained by environmental dependency, cache area vulnerability, reef technology requirements, and logistical demands. By carefully addressing these challenges—particularly through validated artificial reef technologies like the MFAR—the method can serve as a powerful tool within the broader portfolio of coral restoration strategies worldwide.

## Ethics statements

The project was part of an environmental compensation project, and all permits were issued by local authorities for performing the referred.

## CRediT author statement

**Bruno Welter Giraldes**: Conceptualization, Methodology, Validation, Investigation, Writing - Original Draft, Visualization, Project administration. **Caroline Donahue**: Validation, Formal analysis, Investigation, Data Curation, Writing - Review & Editing, Visualization. **Eduardo Santos Mello**: Methodology, Validation, Writing - Review & Editing. **Hamad S. Al-Mohannadi**: Resources, Writing - Review & Editing, Supervision, Project administration, Funding acquisition. **Syed Faisal Mustafa**: Resources, Writing - Review & Editing, Project administration. **Maryam Abdulla**: Resources, Writing - Review & Editing, Supervision, Project administration, Funding acquisition. **Pedro Range**: Conceptualization, Methodology, Validation, Formal analysis, Investigation, Data Curation, Writing - Review & Editing, Supervision, Project administration.

## Declaration of competing interest

The authors declare that they have no known competing financial interests or personal relationships that could have appeared to influence the work reported in this paper.

## Data Availability

No data was used for the research described in the article.
